# The environmental Kuznets curve for Turkish provinces: a spatial panel data approach

**DOI:** 10.1007/s11356-021-17706-w

**Published:** 2021-11-29

**Authors:** Burhan Can Karahasan, Mehmet Pinar

**Affiliations:** 1 Department of Economics and Finance, Piri Reis University, Istanbul, Turkey; 2 Business School, Edge Hill University, Ormskirk, L39 4QP Lancashire UK

**Keywords:** Air pollution, Environmental Kuznets curve, Turkey, Spatial econometrics

## Abstract

This paper aims to test the existence of the environmental Kuznets curve (EKC) hypothesis using SO2 measurements in Turkish provinces between 2004 and 2019. The existing studies concerning the EKC hypothesis for Turkey either use a country-level analysis or panel data techniques covering provincial data that do not account for the spatial dimension. To account for the spatial dependence and overcome the biases resulting from the existence of such spatial spillovers, this paper combines the traditional panel data methodology with the recent advances in spatial econometrics. Our findings confirm the presence of a non-linear link between regional economic prospects and environmental degradation. However, unlike the core expectations of the EKC hypothesis, our results demonstrate a U-shaped relationship between economic development and SO2 levels. Moreover, these findings are robust to the inclusion of a spatial battery which highlights the existence of regional spillovers. Overall, our results show that the post-2000 epoch calls for a different action plan to mitigate the rising impact of environmental degradation in Turkey.

## Introduction

The negative implications of climate change on the world have led countries to implement national and be part of international policies to mitigate the adverse effects of climate change and promote sustainable development. These concerns also led to extensive literature that examined the relationship between economic growth and environmental degradation. One of the most commonly studied research themes exploring the relationship between economic growth and environmental degradation is the so-called environmental Kuznets curve (EKC). The EKC hypothesis argues that the initial economic growth leads to environmental degradation, and after a specific economic development, economic growth leads to an environmental improvement (see e.g., Grossman and Krueger 1991, 1995; Panayotou 1993), resulting in an inverted U-shaped relationship between economic development and environmental degradation.

The overall effect of economic activity on environmental degradation is split into three main factors by Grossman and Krueger (1991): scale, composition, and technique. The scale effect is related to economic activity, which is usually captured by factors such as GDP, urbanization, trade, and FDI. The increased economic activity is considered to increase environmental pollution. The composition effect considers the shift in the economic structure or the mix of economic structure (see e.g., Butnar and Llop 2011; Liu and Wang 2017; Liobikienė and Butkus 2019). In that case, if a regulation or policy promotes comparative advantage in sectors where the environmental regulation is less strict, such regulation or policy will lead to more pollution. On the other hand, if countries shift more towards the service sector, environmental degradation will be lower. Finally, the technique effect refers to the use of advanced technologies and innovations in production, suggesting that the increased economic activity decreases environmental pollution due to stringent policies or shifting towards better technologies (see e.g., Panayotou 1993; Turner and Hanley 2011). For instance, it is found that trade openness could lead to decreased environmental pollution due to access to expensive high technologies (see e.g., Reppelin-Hill 1999; Al-mulali et al. 2015b; Sarkodie and Strezov 2019a; Murshed 2020). Therefore, based on the theoretical arguments, the increased economic activity (scale effect) could have a direct effect on environmental pollution, but the overall impact could vary based on the indirect effects through composition and technique. These theoretical underpinnings led to an increased investigation of the EKC hypothesis.

The EKC hypothesis has been extensively examined, and the literature used various environmental factors as a proxy for environmental degradation (see e.g., Sarkodie and Strezov 2019b) for a detailed review of the EKC hypothesis). Some of the common proxies for the environmental degradation used by the existing studies are CO_2_ emissions (see e.g., Alvarez-Herranz et al. 2017; Apergis 2016; Balsalobre-Lorente et al. 2018; Churchill et al. 2018; Inglesi-Lotz and Dogan 2018; Lau et al. 2019; Sinha and Shahbaz 2018; Yao et al. 2019; Zoundi 2017), ecological footprint (see e.g., Ozcan et al. 2018; Ulucak and Bilgili 2018; Destek and Sarkodie; Dogan et al. 2020), sulfur dioxide (Fang et al. 2020; Grossman and Krueger 1995; He and Lin 2019; Xu 2018; Zhou et al. 2017), water quality (e.g., Fang et al. 2020), and particulate matter concentrations (Ding et al. 2019; Wu et al. 2018; Tirgil et al. 2021), among other environmental factors. Even though most of these studies found support for the inverted U-shaped relationship between GDP per capita and environmental proxy (e.g., Apergis 2016; Destek and Sarkodie 2019; Fang et al. 2020; He and Lin 2019; Sinha and Shahbaz 2018), some studies either found a no support for the EKC hypothesis (e.g., Al-Mulali et al. 2015a; Inglesi-Lotz and Dogan 2018; Ozcan et al. 2018) or found support for an N-shaped relationship between GDP per capita and environmental proxy (e.g., see e.g., Alvarez-Herranz et al. 2017; Balsalobre-Lorente et al. 2018; Churchill et al. 2018; Grossman and Krueger 1995; Zhou et al. 2017).

Energy consumption has been one of the factors used in the analysis as energy consumption has been linked with economic growth (see e.g., Ozturk and Acaravci 2010 for Turkey; Belke et al. 2011, Inglesi-Lotz 2016, and Gozgor et al. 2018 for OECD countries; Bhattacharya et al. 2016 for 38 countries; Chen et al. 2020a for 103 countries; Rahman and Velayutham 2020 for South Asian countries; Ozcan and Ozturk 2019 for 17 emerging countries). Furthermore, energy consumption has also been integrated as part of the EKC hypothesis literature as a control variable while investigating the existence of the EKC hypothesis (see e.g., Al-Mulali et al. 2015a*,* for Vietnam; Destek and Sarkodie 2019 for 11 newly industrialized countries; Sarkodie and Ozturk 2020 for Kenya; Shahbaz et al. 2020 for Chinese provinces; Usman et al. 2019 for India).

The effect of urbanization on emissions has been long examined where the findings on the effects of urbanization on pollution are mixed. Martínez-Zarzoso and Maruotti (2011) found an inverted U-shaped relationship between urbanization and CO2 emissions for 93 developing countries (see e.g., Wang et al. 2015 and Zhang et al. 2017 for similar findings for OECD and 141 countries, respectively). On the other hand, Rafiq et al. (2016) found an insignificant effect of urbanization on emissions, but urbanization led to increased energy intensity in 22 emerging economies (see also Sadorsky 2014 for an insignificant effect of urbanization on CO2 emissions). On the other hand, Liu and Bae (2018) found a positive and significant effect of urbanization on CO2 emissions for China (see also Pata 2018b and Salahuddin et al. 2019 and Solarin et al. 2017; Wang et al. 2018 for similar findings). The abovementioned literature mostly considers the effect of urbanization on CO2 emissions, but the number of studies examining its effect on other air quality measures is limited (see e.g., Ding et al. 2019; Du et al. 2019; Wang et al. 2017b; Xu et al. 2019; Zhou et al. 2018 for the effect of urbanization on PM2.5).

Most of the existing literature either rely on a panel country-level data analysis or time series analysis on a specific set of countries; however, studies at disaggregated data at a national level are quite limited and mostly for China (e.g., Chen et al. 2020b; Ding et al. 2019; Fang et al. 2020; Hao et al. 2016; Shahbaz et al. 2020). Furthermore, most of the existing studies analyzing the EKC hypothesis consider the effect of urbanization and energy consumption on CO2 emissions, and the impact of urbanization on the other air quality factors is limited. Moreover, very few studies control for the possibility of spatial dependence, which may stem from regional economic prospects and local air pollution (e.g., Wang et al. 2013; Li et al. 2019). Therefore, we aim to fill this gap in the literature by using spatial econometrics techniques to account for spatial dependence to examine the relationship between economic development and environmental degradation across Turkish provinces between 2004 and 2019. As an environmental proxy, we will use sulfur dioxide (SO2) and also account for the variation in energy consumption. On the side of regional economic development, we will consider per capita income and urbanization levels of Turkish provinces.

Turkey has experienced major economic growth over the last two decades and is considered to be one of the fastest-growing emerging economies (see e.g., Nathaniel et al. 2020; Petrović-Ranđelović et al. 2020). The gross domestic product (GDP) per capita of Turkey has doubled from $7687 in 2001 to $15,125 in 2019, and during the same period, the percentage of the population living in urban areas increased from 65 to 76, and the population density, measured as people per square kilometer of land area, increased from 83 to 108 (World Bank 2021). The economic growth experienced during this period is also coupled with the increased energy consumption, from 57.84 million tonnes of oil equivalent (Mtoe) in 2000 to 102.96 Mtoe in 2018 (International Energy Agency 2021). Even though the country experienced significant economic growth and urbanization during the last two decades, economic growth shows significant spatial variability across Turkish provinces (Karahasan 2020). There are substantial economic and structural differences across Turkish provinces. For instance, the GDP per capita levels in Ağrı and Istanbul are $2946 and $15,285, respectively (Turkstat 2021). Furthermore, the urbanization, public expenditure, and infrastructural quality levels show major variation across Turkish provinces (see e.g., World Bank 2015). This remarkable regional disparity translates into a persistent duality, which leaves the eastern regions underdeveloped compared to their western counterparts (Doğruel and Doğruel 2003). Given the major economic growth experienced during the last two decades and significant disparities across Turkish provinces, this paper investigates the EKC hypothesis using a local pollution proxy across Turkish provinces while accounting for energy consumption and energy intensity.

There are various ways that this paper contributes to the literature. Firstly, the majority of the existing studies concerning EKC in Turkey employ country-level data using a different set of methodologies (see e.g., Bölük and Mert 2015; Katircioğlu and Katircioğlu 2018; Katircioğlu and Taşpinar 2017; Ozcan et al. 2018; Pata 2018a, 2019; Tutulmaz 2015). In this paper, we aim to examine the EKC hypothesis in Turkey at the provincial level, which could overcome the potential aggregation bias (see e.g., Xu 2018). Xu (2018) demonstrated that the EKC hypothesis found for aggregated data for China is not supported at the provincial level. Secondly, to our knowledge, only two existing studies examine the EKC hypothesis at the provincial level by using panel data estimation techniques (Akbostancı et al. 2009; Tirgil et al. 2021). However, both studies ignore the potential spatial dependence, which would lead to biased results. The biased results in the presence of spatiality have been highlighted recently while examining EKC (see e.g., Hao et al. 2016; Ding et al. 2019). On the other hand, recent papers also started taking into account the cross-sectional dependence (see e.g., Dogan and Seker 2016; Churchill et al. 2018) as the estimations will suffer from size distortions if such dependence exists (Pesaran 2015). To account for the spatial dependence, we will use spatial econometrics techniques and examine the EKC hypothesis in Turkish provinces covering the period between 2004 and 2019. Finally, while other studies consider the effect of urbanization and energy consumption on air pollution, these studies utilize country-level data and consider CO2 emissions per capita as a proxy for air pollution (see e.g., Cetin et al. 2018; Dogan 2016; Ozatac et al. 2017; Pata 2018b; Kirikkaleli and Kalmaz 2020).

The remainder of this paper is organized as follows. The next section provides the detailed variables used in this study and data sources. The methodological approach that incorporates the spatial econometrics method used in this paper is offered in “Methodology” section, and “Results” section provides the empirical findings. Finally, “Conclusions and policy recommendations” section concludes and discusses policy recommendations.

## Data

This study aims to explore the link between regional economic prospects and environmental degradation. For environmental degradation, we use provincial data on sulfur dioxide (SO2).^1^ The dataset covers the 2004–2019 period. For the pre- and post-2007, the data is provided from the Ministry of Health (Moh 2007) and the Ministry of Environment and Urbanization (Meu 2021), respectively. An alternative proxy to account for air pollution is particulate matter (PM10). However, an important concern of the air pollution data is the lack of data for some sample years. Our preliminary check shows that SO2 data has fewer missing values. Therefore, we prefer to use the SO2 measure as the main proxy to account for environmental degradation for the 81 Turkish regions.

To account for the regional economic prospects and development, we use per capita GDP and population density. Revisiting the possible negative externality arguments of agglomeration economies (e.g., Wang et al. 2017a; da Schio et al. 2019), we argue that urbanization is a robustness battery for conditional models and stands as an important determinant that could potentially influence air pollution. While per capita GDP controls for the extent of regional prosperity and wealth, population density acts as a proxy to understand the urbanization level of the provinces. Finally, we control for the provincial per capita electricity consumption. All exogenous variables are at the provincial level (NUTS III) and supplied by the Turkish Statistical Institute for the 2004–2019 period (Turkstat 2021).

We provide complete descriptive statistics in Table 1. Note that Table 1 includes information at the NUTS III level. However, as we will mention in the methodological discussions, we will use data at the NUTS II level in some instances. We provide additional information for the data set at different spatial layers in Appendix Table 5. Our preliminary observations reveal that during the post-2000s, there was a rise in the average per capita GDP, urbanization, and provincial electricity consumption in Turkey. This process corresponds to a fall in the average SO2 levels of the Turkish regions. On the other hand, variation (measured by the coefficient of variation—CoV) of the per capita GDP, urbanization and electricity consumption decline during the post-2000s suggesting an improvement in the spatial disparities. However, during the same period, there was a rise in the variation of the SO2 levels across the Turkish regions. These preliminary findings underline that, despite an average improvement in environmental degradation, the post-2000s period corresponds to spatial instabilities considering the distribution of air pollution. Moreover, the asymmetric path of the regional economic prospects and environmental degradation (rising economic development that matches with falling air pollution) calls for more in-depth analyses of the link between local economic conditions and air pollution.

**Table 1:** Descriptive statistics

	SO2		Per capita GDP		Population density		Per capita electricity consumption	
	2004	2019	2004	2019	2004	2019	2004	2019
Mean	66.282	13.029	6473.859	39,595.500	106.816	132.123	1.538	2.877
Std. dev	31.930	8.888	2621.293	13,645.450	254.152	333.329	1.330	1.662
CoV	0.482	0.682	0.405	0.345	2.379	2.523	0.865	0.578
Min	16.000	3.540	2791.742	16,727.420	10.923	11.084	0.309	0.846
Max	162.000	58.000	14,794.760	86,798.450	2292.289	2986.772	8.050	8.249
N. of obs	49	80	81	81	81	81	81	81

## Methodology

Our baseline specification is a fixed effect panel data model (Eq. 1).^2^ In our empirical analyses, we estimate both unconditional and conditional variants of the model. For each period *t* = 1,2,….*T* and for each province *i* = 1, 2….0.81, we construct panel data to examine the EKC hypothesis in Turkey as follows:

1 $$ln {y}_{it}=\alpha +\beta ln{x}_{it}+\delta ln{d}_{it}+\theta {(ln{x}_{it})}^{2}+\phi {(ln{d}_{it})}^{2}+\gamma ln{e}_{it}+{v}_{i}+{u}_{it}$$

where *y* refers to provincial SO2, *x* is per capita GDP, *d* is the population density and finally, *e* refers to the per capita electricity consumption. *v* is the province fixed effect and *u* is the residuals. Note that, to control for the possible non-linearity within the EKC hypothesis, we also include the squared term for the per capita GDP and population density.^3^

While Eq. 1 controls for the time-invariant heterogeneities, it fails in controlling for the possible spatial mechanisms. As a preliminary check, we implement the spatial auto-correlation analysis using Moran’s I and Geary’s C statistics (Eqs. 2 and 3, respectively).

2 $${I}_{i}=\frac{n}{s}\frac{\sum_{i}\sum_{j}{w}_{ij}({x}_{i}-\overline{x })({x}_{j}-\overline{x })}{\sum {({x}_{i}-\overline{x })}^{2}}$$

3 $${C}_{i}=\frac{(n-1)\sum_{i}\sum_{j}{w}_{ij}({x}_{i}-\overline{x })({x}_{j}-\overline{x })}{2(\sum_{i}\sum_{j}{w}_{ij}{\left({x}_{i}-\overline{x }\right)}^{2})}$$

where *n* represents the number of cross sections and *s* is the summation of all elements in the preferred weight matrix (*w*). Among all possible variants of weight matrices, an inverse distance weight matrix is used in all spatial analyses.

Both statistics test the null hypothesis of spatial randomness. Moran’s I value ranges between 1 and − 1, while values higher and lower than 0 represent positive and negative spatial autocorrelation, respectively. In the meantime, Geary’s C values that are lower (higher) than 1 represent increasing positive (negative) spatial autocorrelation. For Geary’s C and Moran’s I, 0 and 1 represent spatial randomness, respectively.

An important challenge of the spatial analyses is constructing a balanced panel with no missing observations. However, as mentioned before, SO2 measure contains missing values for specific regions. To cope with this problem, we decide the collapse of the provinces (NUTS III regions) into a relatively more aggregate administrative layer (NUTS II regions). While this enables us to decrease the number of missing values, we still observe a limited number of missing values for specific areas. These missing values are filled by interpolating historical data that ranges from 1990 to 2020. While we acknowledge the limitation of interpolating and collapsing the data, it stands alone as the only alternative to moving towards the spatial analyses of the benchmark specification. Furthermore, we provide some descriptive spatial checks to build a safeguard for the empirical approach of the spatial analyses (see “Results” Sect. 4).

Our final specification is a spatial fixed effect panel model (Eq. 4). We allow for three main specifications in our spatial setting. When *λ* = 0, Eq. 4 is defined as a spatial lag model (also known as a spatial autoregressive model—SAR), which defines spatial spillovers over the dependent variable. On the other hand, if *ρ* = 0, then spatial specification will be a spatial error model (SEM) that provides evidence on the spatiality of the omitted variables (thus commons shocks). Note that if both *λ* and *ρ* are different from zero, our specification will be a generalized spatial autocorrelation (SAC) model, which combines the spatiality of the dependent and omitted variables.^4^ To compare the spatial models with the non-spatial variant, we provide a Wald test, which tests the joint significance of the defined spatial mechanisms (Elhorst 2010).

4 $${ln y}_{it}=\alpha +\rho W{y}_{it}+\beta {lnx}_{it}+\delta {ln d}_{it}+\theta ln{x}_{it}^{2}+\phi ln{d}_{it}^{2}+\gamma ln{e}_{it}+{v}_{i}+\lambda W{u}_{it}$$

## Results

Our data set covers the 81 provinces of Turkey for the period of 2004–2019. As SO2 data have missing observations, our baseline non-spatial panel models will be unbalanced. We start by estimating unconditional models, where we only control for the regional economic prospects (Table 2).

**Table 2:** Non-spatial fixed effect panel models

	(1)	(2)	(3)	(4)	(5)	(6)	(7)	(8)	(9)	(10)
GDP per capita	− 0.802***	− 39.232**			− 0.940***	− 6.140***			− 0.942***	− 6.751***
	(0.055)	(16.645)			(0.147)	(1.318)			(0.164)	(1.573)
GDP per capita^2^		3.797**				0.267***				0.303***
		(1.744)				(0.066)				(0.082)
Population density			− 4.734***	0.528			− 2.446***	− 2.052	0.026	− 2.235
			(0.542)	(9.819)			(0.673)	(3.167)	(0.604)	(2.113)
Population density^2^				− 1.450				− 0.041		0.131
				(1.860)				(0.309)		(0.236)
EC per capita					0.345	0.563	− 1.067***	− 1.071***	0.346	0.577
					(0.377)	(0.372)	(0.285)	(0.293)	(0.382)	(0.395)
Observations	1,079	1,079	1,079	1,079	1,079	1,079	1,079	1,079	1,079	1,079
*R*-squared	0.364	0.385	0.165	0.169	0.368	0.391	0.267	0.267	0.368	0.395
Cross sections	81	81	81	81	81	81	81	81	81	81

Robust standard errors in parentheses (clustered at NUTS 3), ***p < 0.01, **p < 0.05, *p < 0.1

Columns 1 to 4 of Table 2 report the unconditional models where we only control for the impact of regional GDP and population density on the SO2 levels. Our initial findings show that both per capita GDP and population density negatively influence SO2 levels. However, the square of the per capita GDP generates a significant positive coefficient suggesting the existence of a non-linear relation. Note that when we control for the possible non-linear impact of population density, we end up with no relationship between urbanization and SO2 level. Next, columns 5 to 8 report the results when we also control for the regional per capita electricity consumption. Our results from these conditional models are virtually unchanged. Electricity consumption has a significant negative influence only in columns 7 and 8 when we control for urbanization. In the remaining conditional models, once the regional income per capita is accounted for, the electricity consumption is no longer significant. Finally, columns 9 and 10 report the results when we control for per capita GDP, population density, and per capita electricity consumption in the same setup. Once again, the impact is non-linear, suggesting that after a certain threshold rising economic prospects (measured by the per capita GDP) will have an adverse effect on air pollution. Note that the explanatory power of the models, which can be examined over the goodness of fit measure (*R*-squared), is highest for the models that control for the regional differences in per capita GDP (and its square). Models that use population density are able to explain only around 17% of the variation in air pollution. On the other hand, models that include regional differences in per capita income are able to explain approximately 37% of the variation in air pollution. Note that, in column 10, when we control for all factors in our empirical setup, our model is able to explain the 40% variation in regional air pollution.

To estimate the spatial variants of the baseline models, we first check for the existence of spatial autocorrelation. We use an inverse distance weight matrix in the spatial analyses.^5^Table 3 provides the results of two separate spatial auto-correlation tests. As mentioned in the previous section, all spatial analyses are carried out at the NUTS II level due to data concerns. Interestingly, SO2 levels for selected years are distributed randomly. On the contrary, per capita GDP, population density, and electricity consumption are spatially autocorrelated. The spatial autocorrelation detected in the income, urbanization, and electricity consumption highlighting the importance of spatiality and clustering behavior in the data. Moreover, this underlines that the economic environment in a region cannot be analyzed separately from its spatial proximity. We also have to note that these findings align with the prior literature that demonstrates the existence of spatial inertia in the local socio-economic conditions (Karahasan 2020). Finally, note that lack of spatial dependence in the SO2 measures does not necessarily rule out the local spatial dependence in air pollution. Instead, it gives an overall idea about spatial dependence at the global level. To better apprehend the spatiality of the regional links, below, we provide some additional descriptive exercises.

**Table 3:** Spatial auto-correlation test results

	SO2		Per capita GDP		Population density		Per capita electricity consumption	
	2004	2019	2004	2019	2004	2019	2004	2019
Moran’s I	− 0.036	− 0.039	0.298***	0.292***	0.026**	0.036**	0.277***	0.251***
	(0.038)	(0.038)	(0.039)	(0.039)	(0.033)	(0.034)	(0.039)	(0.039)
Gearys C	0.999	0.991	0.649***	0.662***	0.928**	0.919**	0.657***	0.672***
	(0.040)	(0.041)	(0.040)	(0.040)	(0.056)	(0.045)	(0.040)	(0.040)

^***^p < 0.01, **p < 0.05, *p < 0.1

**Fig. 1:**
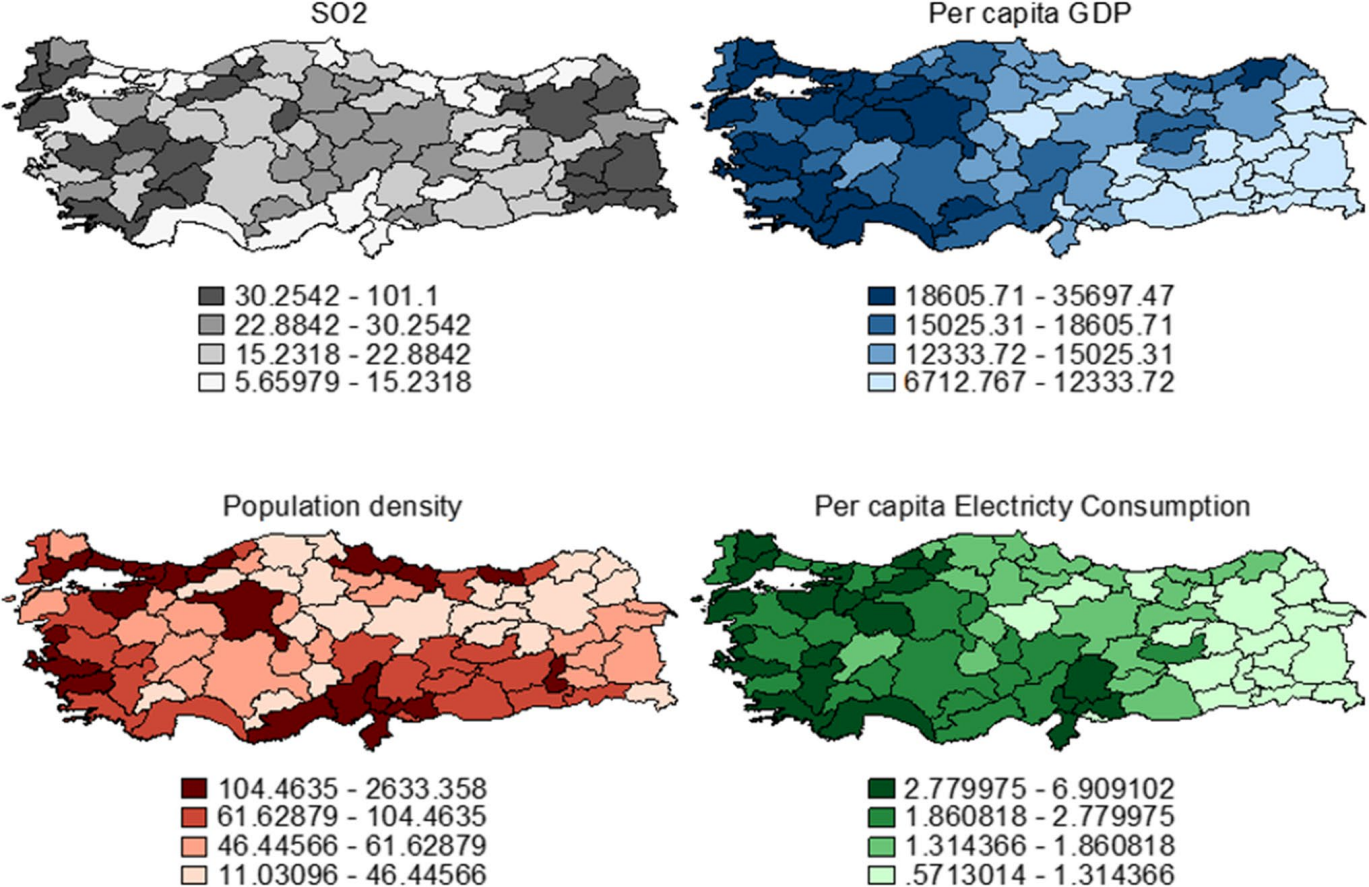
Spatial distribution of variables (NUTS III, 2004–2019 average)

**Fig. 2:**
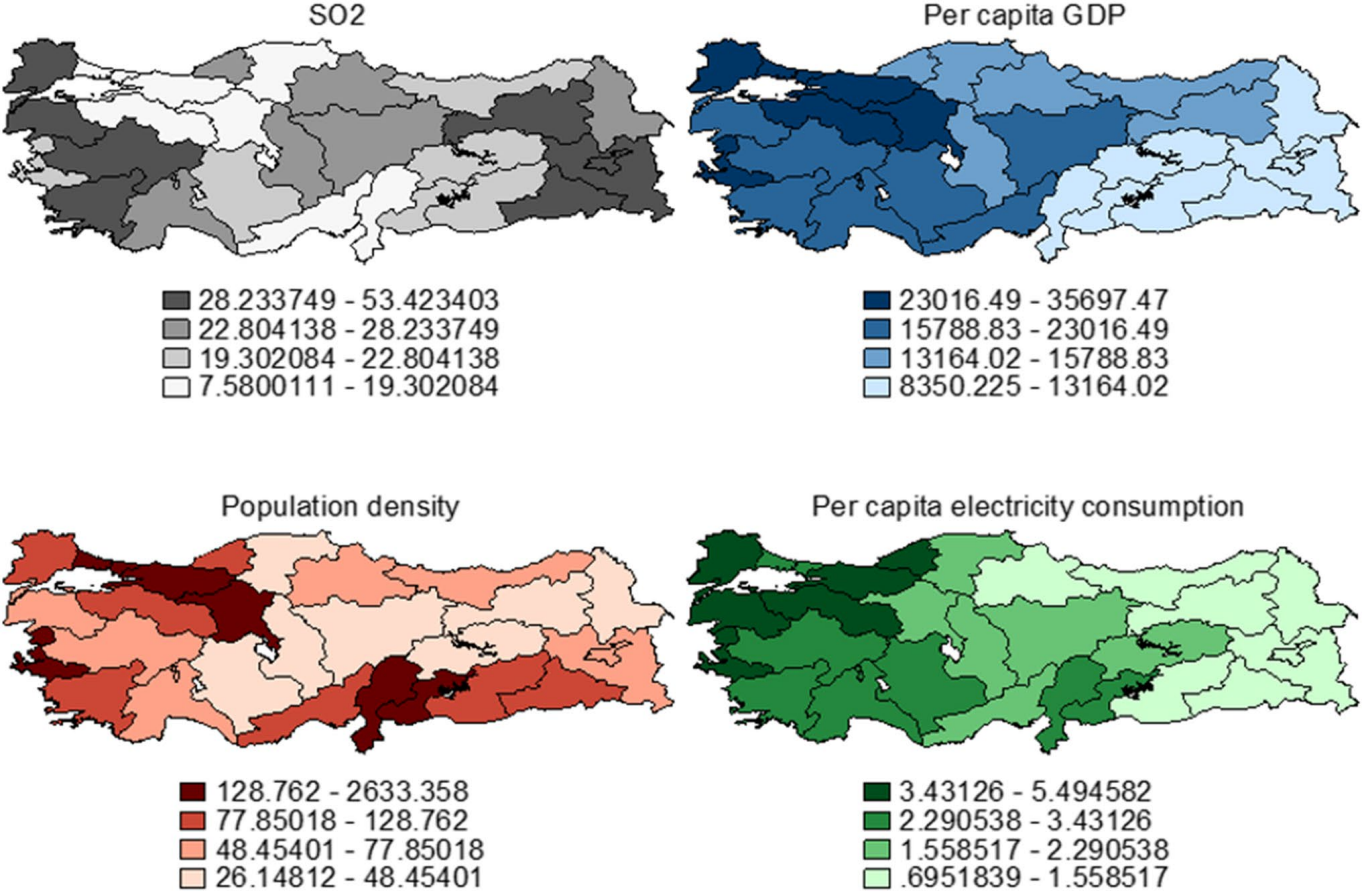
Spatial distribution of variables (NUTS II, 2004–2019 average)

To better understand spatiality, we provide the spatial distribution of the variables for the sample averages. While we lack data for individual years at the NUTS III level, we compute the sample averages and plot the spatial distribution of the variables. Additionally, we provide the spatial dispersion for the same variables at the NUTS II level. Our preliminary comparison shows clear spatial similarity in Figs. 1 and 2, which stands as a safeguard for using NUTS II aggregation in the spatial panel data models. The spatial distribution of per capita GDP perfectly mimics the known developed west and underdeveloped east duality in Turkey. While the western regions of Turkey have an above-average per capita income, southeastern regions are realizing relatively lower regional prosperity. It has to be noted that this pattern is visible at the NUTS III level (Fig. 1). Moreover, once per capita GDP is aggregated at NUTS II level, we observe the continuum of the spatial clustering and polarization among the western developed and eastern underdeveloped regions (Fig. 2). This spatial pattern is also visible in the electricity consumption and population density. We have to highlight that the spatial distribution of the electricity consumption almost perfectly and spatially reflects the income distribution at the NUTS III level (Fig. 1). There is clear evidence for the west–east separation in terms of electricity consumption. Finally, although population density does not mimic the regional separation between developed and underdeveloped regions exactly, it shows the rising urbanization and congestion trends among the developed northwestern regions (Fig. 1) at the NUTS III level. Aggregated NUTS II figures validate that similar urbanization trends at a broader regional separation. It is worth remarking that spatial clustering is extremely persistent for these variables validating our earlier findings on spatial autocorrelation (see Table 2). Additionally, we have to highlight that the spatial distribution of the control variables almost perfectly mimics our prior knowledge on the roots of regional disparities in Turkey (Doğruel and Doğruel 2003). However, it is important to note that even though Moran’s I and Geary’s C measures suggest that SO2 levels are not spatially autocorrelated, there is some spatial clustering at specific locations. For instance, for the SO2 distribution, there is spatial clustering among the eastern and western regions. However, inland areas exhibit a relatively random pattern. We argue that the existence of spatial clustering despite the lack of significant global spatial autocorrelation is vital as it could still jeopardize our initial non-spatial models. Therefore, the spatial variants of the baseline models are also estimated.^6^

We provide the results for the spatially augmented fixed effect panel models in Table 4. Columns 1 to 7 report the results obtained with the conditional SAR model. First of all, our findings show that the coefficient of the spatially lag of the SO2 (*ρ*) is highly significant. The significance of these spatial mechanisms is also validated by the Wald test, which rejects the null hypothesis of spatial randomness. This stands as an important benchmark for the need to consider the spatial dimension. For the overall results, the findings of the SAR models are mostly consistent with the non-spatial panel models. GDP per capita has a negative influence on the regional SO2 level. However, unlike the baseline non-spatial models, non-linear link in the SAR models is relatively weak (only significant at 10% in model 2).

**Table 4:** Spatial fixed effect panel models (SAR, SEM, SAC)

	(1)	(2)	(3)	(4)	(5)	(7)	(8)	(9)	(10)	(11)	(12)	(13)	(14)
GDP per capita	− 0.338***	− 2.085**			− 0.397**	− 1.988*	− 0.953***	− 1.240			− 0.991***	− 0.394	− 1.794***
	(0.121)	(1.008)			(0.170)	(1.170)	(0.163)	(0.896)			(0.193)	(1.250)	(0.716)
GDP per capita^2^		0.086*				0.081		0.015				− 0.033	0.087***
		(0.050)				(0.062)		(0.046)				(0.069)	(0.033)
Population			− 0.616	− 1.253	0.480	− 0.263			0.287	− 1.160	0.338	− 1.932	0.195
Density			(0.665)	(2.024)	(0.912)	(2.320)			(1.334)	(2.569)	(0.895)	(2.453)	(2.102)
Population				0.062		0.034				0.147		0.244	− 0.067
Density^2^				(0.149)		(0.195)				(0.218)		(0.235)	(0.152)
EC per capita	0.092	0.282	− 0.454	− 0.438	0.147	0.292	0.271	0.300	− 0.025	0.048	0.311	0.375	0.116
	(0.421)	(0.444)	(0.288)	(0.305)	(0.442)	(0.467)	(0.436)	(0.454)	(0.596)	(0.628)	(0.464)	(0.472)	(0.281)
*ρ*	0.650***	0.625***	0.695***	0.697***	0.657***	0.628***							0.869***
	(0.076)	(0.072)	(0.077)	(0.077)	(0.077)	(0.076)							(0.028)
*λ*							0.660***	0.656***	0.846***	0.850***	0.662***	0.676***	− 1.815***
							(0.074)	(0.073)	(0.057)	(0.055)	(0.076)	(0.078)	(0.408)
Wald test	73.82***	75.28***	80.56***	81.17***	72.84***	67.55***	78.74***	80.46***	222.05***	236.10***	76.81***	75.58***	1231.99***
	[0.00]	[0.00]	[0.00]	[0.00]	[0.00]	[0.00]	[0.00]	[0.00]	[0.00]	[0.00]	[0.00]	[0.00]	[0.00]
Observations	416	416	416	416	416	416	416	416	416	416	416	416	416
*R*-squared	0.398	0.417	0.252	0.239	0.111	0.350	0.374	0.378	0.066	0.072	0.227	0.042	0.41
Cross sections	26	26	26	26	26	26	26	26	26	26	26	26	26

Robust standard errors in parentheses (clustered at NUTS 3), ***p < 0.01, **p < 0.05, *p < 0.1

We also estimated the SEM variants of the spatial panel models and the results are provided in columns 8 and 13 of Table 4. Once again, spatial battery of the model (i.e., *λ* parameter) is highly significant. Wald test results confirm the applicability of the SEM as a spatial specification. It is worth underlining that in the SEM specifications, impact of per capita GDP is only significant in models 8 and 12, which do not control for the non-linearity. As some of the SEM results do not support the SAR models’ findings, we finally estimate a SAC model that incorporates the spatial mechanisms of the dependent and omitted variables. We found out that both *ρ* and *λ* are statistically significant. Moreover, the Wald test for the joint significance of the spatial batteries points out the need to consider the SAC model. The results from this most augmented spatial specification are reported in column 14 and confirm the U-shaped relationship between GDP per capita and SO2 level.

When we compare the explanatory power of the models, we detect the highest goodness of fit (measured by the *R*-squared) for the models that use the per capita GDP. SAR models that only control for the impact of per capita GDP and its square are able to explain around 40% of the variation in air pollution. On the other hand, explanatory power falls to 24–25% for the SAR models that only control for population density. Similarly, the SEM models that control for the per capita GDP and its square are able to explain 37–38% of the variation in air pollution compared to very low explanatory power when population density is controlled for, approximately 6–7%. Note that for the SAR specification that considers all controls, the *R*-squared value is 35%. While the SEM models with all controls yield very low explanatory power, for the final SAC specification, which controls for two separate spatial channels (spatial lag and error) and the full set of controls, the model’s explanatory power is reported as 41%. Once these findings are compared with the explanatory power of the non-spatial models, we underline the importance of focusing on per capita GDP, controlling for other potential contributors to air pollution (population density and electricity consumption), and use of spatial models to underline the non-linear evolution of air pollution in Turkey.

Overall, our results show that the impact of regional economic prospects on environmental degradation mainly works over per capita GDP distribution. Although population density seems to matter in unconditional models, after controlling for regional income, electricity consumption, and spatiality of the links, the impact of urbanization is found to be insignificant. Our results show that the impact of per capita GDP is robust to the selected spatial specifications. Furthermore, the squared term of the GDP per capita is found to be significant for the SAR and SAC, and these models were the ones that provided the highest explanatory power (measured by the *R*-squared).

In summary, our findings contradict the traditional EKC hypothesis as both the panel and spatial models provided a significant U-shaped relationship between economic development and SO2 levels in Turkey. Our finding is in the lines with the N-shaped relationship between economic development and pollution (see e.g., Grossman and Krueger 1995; Balsalobre-Lorente and Álvarez-Herranz 2016; Allard et al. 2018) where the relationship between economic development and pollution becomes positive because of diminishing returns on technological changes after a certain income level, and the Turkish provinces experiencing the second phase of the N-shaped relationship. Another explanation is that economic growth in the peripheral regions may have been adopting relatively older and obsolete technologies, and the economic growth leading to increased air pollution (see e.g., Destek and Sarkodie 2019), and increased economic growth in these regions may have led to decreased energy efficiency, which then leads to higher air pollution.

## Conclusions and policy recommendations

Environmental degradation is a rising global concern. Cross-country evidence suggests that economic prospects have a pervasive influence on environmental conditions. The so-called EKC hypothesis postulates the possible non-linearity of the impact of economic conditions. Global evidence suggests that the initial impact of economic growth and development is primarily negative, which is expected to be positive after the threshold level income level. However, there is also evidence on alternative non-linear links where either U- or N-shaped relationship between economic prospects and environmental degradation was found.

This paper examines these discussions for a developing country example, Turkey. Given the dual economic structure, we examine the possible links at the regional level. Both panel and spatial estimations point out the existence of a U-shaped relationship between regional economic prospects and air pollution. Our additional analyses call for the importance of using spatial specifications. Among different spatial specifications, our results highlight the validity of various spatial spillovers across the Turkish regions. More remarkably, spatial panel fixed effect models validate the importance and dominance of the asymmetric impact of per capita GDP’s impact on environmental degradation. This finding mostly corresponds to rapid improvement in air quality during the post-2000s, where metropolitan urban areas adopt various measures to decrease air pollution. An important example is a shift from coal-based in-house heating towards natural gas usage. It could partially be linked with the ongoing deindustrialization in historically manufacturing-oriented territories.

This study’s findings contribute to our knowledge on the importance of local economic prospects for understanding the evolution of environmental degradation. Besides, it is one of the first attempts that incorporates the impact of spatiality in the empirical setup testing the EKC for Turkey. Our results also contain information about the possibility of a different non-linear mechanism. Considering the post-2000s, we highlight that air pollution stands as an environmental concern stemming from Turkey’s relatively less developed territory. In the meantime, regions with better economic conditions have a less negative influence on environmental degradation, suggesting that the source of the environmental conflict shifts towards relatively less developed regions of Turkey. This pattern conflicts with the pattern observed in the empirical literature that investigates the pre-2000s. Therefore, changing nature of the local economic prospects and environmental degradation will potentially call for a different action plan for the future. While policy tools of the 2000s in urbanized regions positively influence improvements in air quality, the lack of policy action on the peripheral regions is quite visible in terms of rising concerns on peripheral air pollution. Therefore, to combat with the increased air pollution, governments should promote cleaner technology use in the peripheral areas and also adopt stricter environmental regulations in these regions to avoid potential within-country pollution haven hypothesis (i.e., production moving from developed regions to less developed regions due to lower environmental regulations or auditing).

Inevitably, there are also some limitations of this study. As air pollution data contains missing values, we aggregate the data at the NUTS II level for the spatial analyses. This results in a loss of locality in the empirical analyses. Moreover, our analyses cover the post-2000s. This period corresponds to rapid change and transformation in the Turkish economy. More remarkably, there is a huge spatial reshuffling of income distribution throughout the sample period. One interesting exercise would be to form a historical database for air pollution and local economic prospects and test the same hypothesis for a longer time dimension. However, we could not perform such analyses due to two reasons. First, data quality for air pollution worsens for the pre-2000s with rising missing information at the regional level. Second, provincial GDP data has a methodology change after 2004, which makes the pre- and post-2000s incomparable in an analytical setting. Furthermore, the availability of data on trade and FDI activity at the regional level disallows us to examine the relevance of these factors for the EKC hypothesis. The availability of such data in the future would enrich the discussion and more in-depth analysis of the EKC hypothesis at the regional level. Another important dimension is the use of only air pollution data to understand environmental degradation. However, there are other potential threats to environmental worsening. For instance, water pollution stands as an important challenge for industrializing countries and regions. This could be an important line of research based on higher-quality data on water pollution. While we acknowledge all these limitations, we believe that our findings provide interesting and valuable insight to understand how environmental degradation responds to the changing regional economic prospects during a period of rapid change and transformation in a country with substantial spatial disparities.

## Data Availability

The data that support the findings of this study are available from the Turkish Ministry of Health, the Ministry of Environment and Urbanization of Turkey, and the Turkish Statistical Institute.

## Appendix

**Table 5:** Explanation of the variables

Variable	Source	Number of obs.* at NUTS III	Number of obs at NUTS II
SO2	Ministry of Health (2004–2007)	Number of annual observations vary within a range of 49–78 during the 2004–2007 period [49, 50, 42, 78 respectively]	26 for each year (2004–2019)
	Ministry of Environment and Urbanization (2008–2019)	Number of annual observations vary within a range of 47–81 during the 2008–2019 period [81, 81, 81, 50, 47, 77, 81, 53, 69, 80, 80, 80 respectively]	
Per capita GDP	Turkish Statistical Institute (TurkStat)	81 for each year (2004–2019)	26 for each year (2004–2019)
Population density	Turkish Statistical Institute (TurkStat)	81 for each year (2004–2019)	26 for each year (2004–2019)
Per capita electricity consumption	Turkish Statistical Institute (TurkStat)	81 for each year (2004–2019)	26 for each year (2004–2019)

Pre-interpolation numbers of observations for SO2 at NUTS III level are provided within the parentheses

## Footnotes

1 Provinces correspond to the NUTS III regions in Turkey. For a brief overview of the regional NUTS classifications in Turkey see https://ec.europa.eu/eurostat/documents/345175/7773495/TR.pdf (last accessed on 12.06.2021).

2 A second alternative for panel model specification is the random effect model. However, as fixed effects specification controls for the time-invariant heterogeneities, it will account for Turkey’s persistent dual economic structure (see Doğruel and Doğruel (2003) for a brief overview of the regional heterogeneities in Turkey).

3 A potential augmentation could be to use cubic terms for per capita GDP and urbanization. We also estimate models that include the cubic terms; however, the inclusion of cubic terms led to a drastic fall in the explanatory power of the models. This could be partially due to the short time dimension of the panel, which prevents accounting for the possible cyclical path of the relationships.

4 Another alternative spatial model would be the spatial Durbin model (SDM), which also allows for spatiality in the exogenous variables. While we also estimate SDM, results point out a lack of significant spatial effects from the exogenous variables. These results are available upon request.

5 We also replicate all spatial analyses by using a contiguity weight matrix. Our results are the same and available upon request.

6 Spatial distribution of the variables indicates the possibility of spatial heterogeneities, which would call for a different setup in empirical terms (Anselin 1995). However, due to the low number of cross sections at the NUTS II level (i.e., 26), we skip the more detailed analyses of the spatial heterogeneity. This stands as a potential line of future work conditioned on data availability.
